# Supercritical CO_2_ Extracts for Food Preservation: Efficacy and Interaction with Black Soldier Fly Larvae Fat in Food Matrixes

**DOI:** 10.3390/ijms26199536

**Published:** 2025-09-29

**Authors:** Aelita Zabulionė, Antanas Šarkinas

**Affiliations:** Food Institute, Kaunas University of Technology, LT-50254 Kaunas, Lithuania; aelita.zabulione@ktu.lt

**Keywords:** SC-CO_2_, antimicrobial activity, black soldier fly larvae, food shelf life, marjoram, bay leaf, dashi, cinnamon bark, rosemary, caraway, chamomile, ginger, dried clove bud

## Abstract

This study investigated the antimicrobial efficacy of supercritical carbon dioxide (SC-CO_2_) plant extracts as a natural preservative, prolonging food shelf-life. The research evaluated the performance of 10 different extracts, including cinnamon, thyme, clove, and dashi, in low-fat food matrices. The results showed that these extracts significantly prolonged the shelf life of a plant-based and animal-based matrixes, with cinnamon and dashi extracts proving highly effective in plant-based matrix against mould and yeast growth for up to 65 days. A key part of the study focused on the interaction between these lipophilic extracts and black soldier fly larvae (BSFL) fat as a potential carrier system. While fats were expected to improve the extracts’ sensory properties and act as a delivery system, in vitro tests revealed an antagonistic effect. The lipophilic nature of the extracts’ active compounds caused them to be trapped within the fat phase, rendering them unavailable to interact with pathogens. These findings highlight the challenges and potential of using lipophilic natural antimicrobials in food systems and underscore the need for new strategies to optimize their efficacy.

## 1. Introduction

The food industry is increasingly turning to natural, organic antimicrobial substances due to growing concerns about the environmental and health impacts of synthetic chemicals. Organic compounds derived from botanical extracts, especially those processed with supercritical carbon dioxide (SC-CO_2_), have emerged as a promising, sustainable alternative to combat harmful microorganisms and extend food shelf life. These natural substances offer several advantages, including their plant origin and potential for targeted applications.

SC-CO_2_ extraction is a cutting-edge technique used to obtain bioactive compounds from natural sources. This method utilizes carbon dioxide in a unique supercritical state, where it behaves as both a gas and a liquid. Unlike traditional methods that rely on harsh chemical solvents, SC-CO_2_ extraction is solvent-free. This makes the process environmentally friendly and eliminates the risk of chemical residues in the final product.

The process also yields higher concentrations and greater purity of bioactive compounds, which is crucial for creating potent antimicrobial products. Furthermore, because SC-CO_2_ extraction operates at lower temperatures than conventional methods, it protects heat-sensitive compounds from degradation, ensuring the final extract retains its full bioactivity.

The potential of these botanical extracts in ensuring food safety is vast. Many plant species have been investigated for their natural antimicrobial properties, making them attractive candidates for new food preservation solutions. This research explores how these extracts can be applied to protect food from spoilage while offering a cleaner, safer product for consumers.

Thyme essential oil contains high amounts of thymol and carvacrol, which are lipophilic (fat-soluble) phenolic compounds. These compounds are known to disrupt bacterial cell membranes, which is a key step in their antimicrobial action. The lipophilicity of these compounds allows them to easily penetrate the cell wall of Gram-negative bacteria, which is primarily composed of lipids [1].

Chamomile essential oil contains various lipophilic compounds, including terpenes (highly lipophilic hydrocarbons, for example, bisabolol oxides A and B, chamazulene, and farnesene) and flavonoids (although not as lipophilic as terpenes, some chamomile flavonoids possess lipophilic properties due to their aromatic ring structures) [2].

Bay leaves are rich in essential oils that contain various lipophilic compounds, primarily terpenes such as 1,8-cineole (eucalyptol), α-pinene, myrcene, and linalool. These terpenes are highly lipophilic due to their hydrocarbon nature. Study [3] emphasizes that the lipophilic nature of these compounds plays a crucial role in their antimicrobial activity. They can easily penetrate lipid-rich bacterial cell membranes, disrupting their integrity.

Dashi essential oil is rich in lipophilic compounds, primarily terpenes, with carvacrol and thymol being the main components. These phenolic monoterpenes are highly lipophilic due to their hydrocarbon structures. Study [4] emphasizes the role of these lipophilic compounds in the antimicrobial activity of dashi essential oil, particularly their ability to disrupt bacterial cell membranes.

In this study [5], the antimicrobial effect of rosemary essential oil on foodborne pathogens is investigated. Rosemary essential oil is rich in lipophilic compounds, primarily terpenes such as: 1,8-Cineole, α-pinene, and camphor. These terpenes are highly lipophilic due to their hydrocarbon nature. The study emphasizes that these lipophilic compounds contribute to the antimicrobial activity of rosemary essential oil by disrupting bacterial cell membranes.

Another article [6] discusses the antimicrobial properties of cinnamon. The main active compound in cinnamon responsible for its antimicrobial effect is cinnamaldehyde. Cinnamaldehyde is a lipophilic compound due to its aromatic structure. This lipophilic nature allows it to easily penetrate bacterial cell membranes, disrupting their integrity and causing cell death.

In this study [7], the chemical composition and antimicrobial activity of marjoram essential oil are analyzed. Marjoram essential oil is rich in lipophilic compounds, primarily terpenes, with carvacrol and thymol being the main components. These phenolic monoterpenes are highly lipophilic due to their hydrocarbon structures.

Ginger essential oil is rich in lipophilic compounds, primarily terpenes, such as: zingiberene, β-bisabolene, and α-farnesene. These terpenes are highly lipophilic due to their hydrocarbon nature and disrupt the function of bacterial cell membranes [8].

Carraway essential oil is rich in lipophilic compounds, primarily terpenes, with cuminaldehyde being the main component. Cuminaldehyde is a highly lipophilic compound due to its aromatic structure. Study [9] emphasizes the role of these lipophilic compounds, especially cuminaldehyde, in the antimicrobial efficacy of carraway essential oil.

All these findings show that the selected plant materials have very important lipophilic compounds that are believed to possess antimicrobial activity. But to expand their applicability in industry new approaches are needed. Due to their exceptional sensory properties, although they have significant antimicrobial efficacy, SC-CO_2_ extracts are used relatively rarely, especially in the food industry.

Since supercritical carbon dioxide (SC-CO_2_) extracts are lipophilic, they can be easily mixed with edible fats. This not only helps them blend into food products but also improves their sensory properties. Most flavour and aroma molecules are fat-soluble, which means they can be trapped within saturated fats. This helps to control the release of these compounds, preventing a strong, immediate burst of aroma and flavour that might be overpowering.

Instead of a quick sensory experience that fades fast, the fat matrix enables a slow, continuous release of flavour as the food is consumed and warmed. This controlled release can result in a more complex and prolonged sensory experience. Essentially, the fat does not destroy the flavour molecules; it simply alters their bioavailability and the rate at which they are perceived. This suggests that incorporating extracts into fats could improve their usefulness and technological potential in the food industry.

Although many fats could be used, black soldier fly larvae (BSFL) fat was chosen for this study. While public opinion on the use of BSFL for food remains cautious, this fast-growing, undemanding, and valuable biomass can offer greater stability and security for local economies in times of uncertainty.

Although BSFL proteins are the most researched component, the larvae also produce a significant amount of fat. This makes it crucial to find meaningful and consumer-friendly applications for BSFL fat in food, potentially by helping products stay edible and safe for longer. A previous study found that BSFL fat is highly stable and has a slight antibacterial effect, which led to the decision to explore its potential further.

Given that BSFL fat has relatively low antimicrobial efficacy on its own, the aim of this study was to test its interaction with lipophilic extracts from various herbs and plants. The goal was to determine if a synergistic interaction could be found. This research represents an effort to create a new functional food component by combining two underutilized raw materials with significant potential.

Both BSFL fat and strong plant extracts face consumer acceptance challenges: BSFL fat has negative psychological associations, while the intense taste and smell of plant extracts make them difficult to use without spoiling the final product’s sensory profile. The core objective of this work was to find a combination of these two substances that would contain a small amount of plant extract (to improve sensory properties) while still providing a strong antimicrobial effect.

## 2. Results

The obtained plant extracts were analyzed within 10 days of production to determine their microbial contamination and chemical composition before being directed to laboratory efficacy tests.

More information about BSFL fats can be found in our previous research [10] and for the sake of clarity BSFL fat composition is given in mg/g of fat: lauric acid—464.2, palmitic acid—154.0, oleic acid—140.2, myristic acid—73.1, linoleic acid—68.0, palmitoleic acid—53.6, stearic acid—18.2, capric acid—11.8, myristoleic acid—3.4, heneicosanoic acid—1.8, arachidic acid—0.8, pentadecainoic acid—0.3, margaric acid—0.3, gadoleic acid—0.2, elaidic acid—0.1.

### 2.1. Characterization of Plant Extracts Fatty Acids Composition

Since carbon dioxide is a non-polar solvent, a significant portion of the plant extracts obtained with it consists of lipids. Different fatty acids have different properties, so their fatty acid analysis can provide valuable information for accurately predicting the potential of the resulting extracts. Table 1 presents a detailed fatty acid profile of all plant extracts, and below, we discuss the fatty acids found in significant quantities (>10% of the total fat content) in the extracts.

Hexanoic acid (C6:0), also known as caproic acid, has antimicrobial properties against various microorganisms, including bacteria, fungi, and yeasts. Hexanoic acid is considered a short-chain fatty acid, and its antimicrobial effect is related to its ability to disrupt microbial cell membranes. Hexanoic acid can alter the integrity and permeability of microbial cell membranes, leading to cell leakage and eventual death. Hexanoic acid can also lower the pH of the surrounding environment, creating an acidic medium that inhibits the growth of many microorganisms [11]. Hexanoic acid was also found in the studied rosemary plant extract, 212.6 mg per gram of extract.

Decanoic acid (C 10:0), also known as capric acid, is a medium-chain fatty acid with antimicrobial properties. A study [12] showed that it can inhibit the growth of various microorganisms, including bacteria, fungi, and yeasts. The known mechanisms of action include:

1. Damage to cell membranes. Decanoic acid can disrupt the integrity of microorganism cell membranes, which can lead to cell lysis and death.
2. Disruption of cellular metabolism. It can interfere with essential cellular processes such as protein synthesis and energy production.

Decanoic acid has been shown to have a direct antimicrobial effect on many microorganisms, including *Staphylococcus aureus*, *Escherichia coli*, *Candida albicans*, and *Aspergillus niger*. The antimicrobial action of a SC-CO_2_ plant extract is a result of multiple mechanisms working together. For example, a THEx and DCFBEx extracts have significant amounts of the capric acid (41.3 and 184.1 mg/g extract, respectively). Capric acid disrupts the membrane and can interfere with cellular metabolism.

A study [13] found that undecylic acid (C 11:0), together with lauric acid and *N*-tridecanoic acid, effectively inhibits the formation of persister cells in *E. coli*. Persister cells are a subpopulation of bacteria that are highly resistant to environmental stresses and even to antibiotics. The study also showed that these fatty acids inhibit *E. coli* biofilm formation. Another study [14] found that undecylic acid has antifungal properties, particularly against fungal infections such as *C. albicans*. Undecylic acid was also found in the marjoram plant extract at 59.3 mg/g extract.

Hexadecanoic acid (C16:0), or palmitic acid, also has antimicrobial effects. Palmitic acid is a long-chain saturated fatty acid with antimicrobial properties. The antimicrobial activity of palmitic acid is primarily linked to its ability to disrupt the integrity of microbial cell membranes. It can integrate into the bacterial membrane, altering its permeability, which eventually leads to bacterial cell death [15]. Palmitic acid was found in all the extracts tested, but was most abundant in the ginger extract at 57.2 mg/g extract.

Oleic acid (C18:1) is a fatty acid naturally found in various animal and plant fats and oils. Oleic acid has been found to have an antibacterial effect, particularly in inhibiting the growth of Gram-positive bacteria species. Oleic acid was also found in the studied rosemary, ginger, chamomile, and carraway extracts, constituting 62.9, 48.7, 76.0, and 215.4 mg/g extract, respectively.

Linoleic acid (C18:2) is an essential omega-6 fatty acid with antimicrobial properties against certain microorganisms. Studies [16] have shown that linoleic acid can inhibit the growth of some Gram-positive bacteria, such as *S. aureus* and *B. subtilis*. Linoleic acid was found in all the extracts tested, but the most significant amounts were found in the cinnamon, carraway, dashi, and ginger plant extracts, constituting 408.8, 119.5, 175.7 and 56.3 mg/g of extract, respectively.

Linolenic acid (C18:3a) is an essential omega-3 fatty acid that possesses antimicrobial properties against certain microorganisms and has a very similar mechanism of action and spectrum as linoleic acid. Linolenic acid was also found in the studied marjoram, bay leaf, dashi, and thyme plant extracts, constituting 54.2, 170.5, 384.7 and 95.9 mg/g of extract, respectively.

In the chamomile extract, γ-linolenic acid (C18:3γ) was found (264.5 mg/g of extract). Some studies have shown that γ-linolenic acid may have an antimicrobial effect on certain microorganisms. Studies [17] have shown that γ-linolenic acid can inhibit the growth of some oral pathogens, for example, those associated with gingivitis.

### 2.2. Sterility Analysis of Plant Extracts

To create a product with a long shelf life, tests for the bacterial contamination of the resulting plant extracts were performed. The extracts were tested within 10 days of production.

To determine the bacterial contamination of the extracts, the following tests were conducted: total microbial count, count of beta-glucuronidase-producing *E. coli*, detection of coliform bacteria, count of enterobacteria, count of presumptive *B. cereus*, count of coagulase-positive staphylococci, count of *L. monocytogenes*, detection of *Salmonella*, count of proteolytic bacteria, count of yeasts, and count of moulds. In none of the samples did any of the tested indicators exceed the detection limit (i.e., all results were less than 1 CFU or not detected). Detailed data from the bacterial contamination tests of the extracts are provided in Appendix A Table A3.

### 2.3. Antibacterial Activity of Extracts and Their Mixtures

Due to the dense nature of the plant extracts, a solvent or carrier was required to test them at low concentrations. For initial antibacterial activity tests, the extracts were first dissolved in ethanol. This allowed for an even distribution of the sample at low concentrations. Since ethanol evaporates rapidly, it was assumed that the observed efficacy and resulting inhibition zones were solely due to the properties of the extract itself.

A control plate, containing the test culture treated only with ethanol, was used for each measurement to ensure the results were accurate. Once the minimum inhibitory concentrations (MIC) were determined using the ethanol solution, these values were used to guide further studies with BSFL fat. The consistency of this method ensured a reliable baseline for comparison. Table 2 shows the results of MIC determination. Table A1 additionally shows the inhibition coefficients achieved for each concentration and each extract used.

Thyme extract showed exceptional activity against all tested microorganisms, thus confirming the hypothesis that it is possible to create a very broad-spectrum component capable of increasing food safety and inhibiting pathogen growth. It is also important to note the exceptional activity of thyme extract against *L. monocytogenes*. This demonstrates its huge potential for creating innovative component, or even surface disinfectants for the food industry.

To optimize the ongoing study, the antibacterial efficacy of plant extracts was determined against reference microorganisms. The selected microorganisms are commonly found in plant-based food products, possess specific characteristics, and are registered in international microorganism banks.

1. *E. coli* (ATCC 8739), a Gram-negative bacterium that stains red and is a facultative anaerobe, was chosen to represent this group.
2. *S. aureus* (ATCC 25923), a Gram-positive coccus that stains blue, was chosen to represent this group.
3. *L. monocytogenes*, a Gram-positive bacterium that stains blue, is a facultative anaerobe, and grows at low temperatures, was also included.

#### 2.3.1. Antibacterial Activity of Extracts in Ethanol

**Figure 1:**
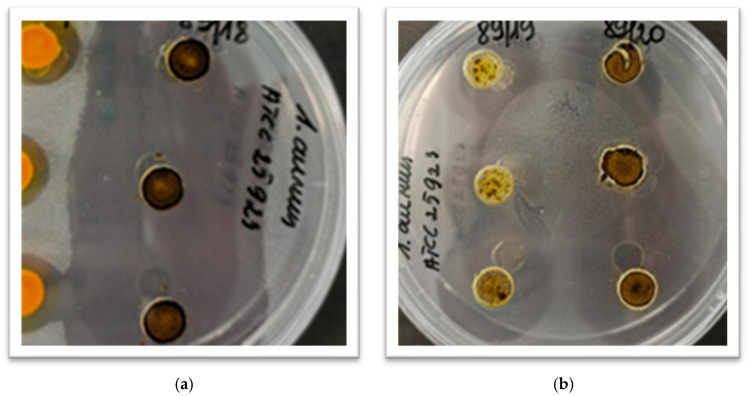
Examples of inhibition zones for microorganisms treated with plant extracts. The figure shows: (**a**) *S. aureus* treated with dashi extract; (**b**) *S*. *aureus* treated with bay leaf extract (89/19) and marjoram (89/20) extract.

Antimicrobial activity was quantified by calculating the inhibition coefficient, which is based on the diameter of both the initial well and the resulting inhibition zone. Figure 1 illustrates this, showing that in some cases, the inhibition zones have expanded and merged together. This underscores the importance of testing a wide range of concentrations.

By doing so, we could accurately assess the inhibition coefficient and better understand which extracts have the greatest potential for activity at both low and very low concentrations. This approach was essential for identifying the most effective natural preservatives.

The percentage concentration of solutions indicates the amount of a substance in the solution. 96% ethanol was used as the solvent. High concentrations (75% and 50%) were chosen to determine if a specific extract has activity against the test microorganism. The lower concentrations (25%, 10%, 5%, 1%, 0.5%, 0.25%, 0.125%, 0.063%, 0.0313%, 0.0156%, 0.00781%) were used to identify the lowest extract concentrations that provide a plausible inhibitory effect.

Extracts that showed activity at 1% or higher concentration were not considered to further investigation, because the purpose of the test object is to test possibility to extend the shelf life of food products, and large amounts of additives may be unacceptable to the sensory parameters.

Link between antimicrobial activity and fatty acid composition is particularly relevant for supercritical CO_2_ plant extracts because they naturally contain a significant amount of the plant’s fatty acid profile in addition to the essential oil components. This means that the antimicrobial activity of these extracts is not solely due to the essential oils but is a combined effect of both the terpenes and the fatty acids present. The specific types and concentrations of fatty acids in the extract, therefore, directly influence its overall antimicrobial potential.

The extracts showed a wide range of potencies, with some exhibiting broad-spectrum activity at very low concentrations. RHEx (rosemary herb extract), CBEx (cinnamon bark extract), THEx (thyme herb extract), DHEx (dashi herb extract) and MHEx (marjoram herb extract) showed the highest potential. RHEx demonstrated the highest overall efficacy. It was exceptionally potent against Gram-positive bacteria, with MIC of 0.0156% against *S. aureus* and 0.0313% against *L. monocytogenes*. Its activity against Gram-negative *E. coli* was also strong, with an MIC of 1%. The study [18] showed that rosemary essential oil MIC against *E. coli* 0.625%.

CBEx was also highly effective, particularly against the Gram-positive strains. It showed an MIC of 0.0313% against both *S. aureus* and *L. monocytogenes*, and a respectable MIC of 0.5% against *E. coli*. The study [19] showed that the MIC of cinnamon essential oil against *E. coli* was 0.182% and against *S. aureus* 0.469%. Another study [20] determined cinnamon essential oil MIC against *S. aureus* to be 0.0256% and against *E. coli*—0.1024%.

THEx proved to be a powerful broad-spectrum antimicrobial. It had a MIC of 0.125% against *E. coli* and a very low MIC of 0.0625% against both *S. aureus* and *L. monocytogenes*. Review [21] claims that thyme essential oil concentrations as low as 0.01% and 0.05% effectively reduced growth of *S. aureus* up to 53% and 76%, respectively. DHEx also displayed notable efficacy, especially against Gram-positive bacteria, with a very low MIC of 0.0625% against *S. aureus* and 0.125% against *L. monocytogenes*. Its MIC against *E. coli* was notably high at 5%. Another study [20] determined thyme essential oil MIC against *S. aureus* and *E. coli*—0.0256%.

MHEx was highly effective against *L. monocytogenes* (MIC of 0.0625%) and also showed good activity against *S. aureus* (MIC of 0.25%) but notably higher against *E. coli* (MIC of 5%). The study [22] found that the effective concentration of marjoram essential oil against *S. aureus* was between 0.0156 and 0.0313. Another study [23] found that marjoram essential oil demonstrated significant antibacterial activity. It had a MIC value of 0.125% against several strains, including *S. aureus* ATCC 25923, *S. aureus* MRSA ATCC 43300, and *E. coli* ATCC 25922. Only the *E. coli* AG100 strain showed less susceptibility, with an MIC of 0.250%.

Another group of extracts, including BLEx (bay leaf extract) and DCFBEx (dried clove flower bud extract), showed moderate antimicrobial activity. BLEx had an MIC of 50% against *E. coli* but was more than 100 times more potent against *L. monocytogenes* (0.125%) and *S. aureus* (0.5%). The study [24] showed that bay leaf SC-CO_2_ extract MIC against *S. aureus* 0.128%.

Similarly, DCFBEx had MIC of 0.25% against both *E. coli* and *S. aureus* and 0.125% against *L. monocytogenes*, indicating a consistent, moderate-level of activity. The study [19] showed that clove buds essential oil MIC against *E. coli* was 10%, against *S. aureus* 5%. Another study [20] determined clove buds essential oil MIC against *S. aureus* and *E. coli* to be 0.0512%.

Some of the extracts have shown plausible activity against *S. aureus*, but required very high concentrations to show any activity on other tested microorganisms and for this reason ChFEx (chamomile flower extract), GREx (ginger root extract) and CFEx (caraway fruit extract) are here considered as having low antimicrobial activity. ChFEx, GREx and CFEx were generally less effective, particularly against *E. coli* and *L. monocytogenes*. All three extracts had MIC greater than 75% against *E. coli*, suggesting a low level of activity against this Gram-negative bacterium. However, both ChFEx and GREx showed some potency against *S. aureus* (MIC of 0.125% and 0.0625%, respectively), while CFEx was consistently less effective across all three microorganisms. The study [19] showed that the MIC of ginger essential oil against *E. coli* was 0.052% and against *S. aureus* 0.062%.

The results highlight that certain extracts, particularly RHEx, CBEx, THEx, and DHEx, are promising candidates for natural food preservation due to their ability to inhibit bacterial growth at very low concentrations.

Caproic acid and capric acid fatty acids have high antimicrobial activity, and extracts rich in them (rosemary extract has 212.6 mg caproic acid per gram of extract and clove extract has 184.1 mg of capric acid per gram of extract) have been found to be effective against test microorganisms. These extracts demonstrated high efficacy against test microorganisms—the MIC of rosemary against *E. coli* was 1%, against *S. aureus* 0.0156% and against *L. monocytogenes* 0.0313%. Clove extract also showed promising results against the test microorganisms—the MIC against *E. coli* and *S. aureus* was 0.25%, and against *L. monocytogenes* 0.125%.

Many studies on this topic tend to focus on essential oils, and similar trends are often seen in their antibacterial efficacy compared to SC-CO_2_ extracts due to differences in technology and composition. However, SC-CO_2_ extracts, which are less pure, generally require a higher minimum concentration to inhibit bacterial growth. While this might seem like a drawback, it is a necessary trade-off for more sustainable processes. Evaluating the effectiveness of less-pure products is crucial for developing the most sustainable final solutions.

#### 2.3.2. Antibacterial Activity of Extracts in BSFL Fats

The role of BSFL fats is complex: they can be a barrier that reduces the antimicrobial effect of SC-CO_2_ extracts, but they can also be active components themselves or a functional part of the delivery system for those same compounds. The most plausible interactions between BSFL fats and used SC-CO_2_ extracts is the attenuation or inhibition of the antimicrobial efficacy. This phenomenon can be attributed to the inherent lipophilicity of numerous active constituents found in SC-CO_2_ extracts, such as terpenes and phenolic compounds. While inhibition between different fats, that separately exhibit antimicrobial properties, is common, certain fatty acids can also work with or on their own to enhance antimicrobial effects. Fats and other lipids can act as a carrier for SC-CO_2_ extracts, helping to stabilize volatile compounds and control their release over time. This can potentially prolong their protective effect within a food matrix, which is a key goal in food preservation.

The fats of BSFL are rich in medium-chain fatty acids (MCFAs), most notably lauric acid (C 12:0), which in our case constitute a 464.2 mg/g BSFL fat. MCFAs like lauric acid have potent antimicrobial properties on their own. They work by disrupting the cell membranes of microorganisms, causing them to break down and die. This is particularly effective against Gram-positive bacteria, yeast, and fungi. When BSFL fat combined with SC-CO_2_ extracts it is expected to create a “dual-action” approach that can attack pathogens from multiple angles, leading to a stronger overall effect. In light of these facts, it may appear that the most likely outcome is a synergistic effect of BSFL fat and SC-CO_2_ extracts, but this hypothesis was not confirmed after performed research.

**Figure 2:**
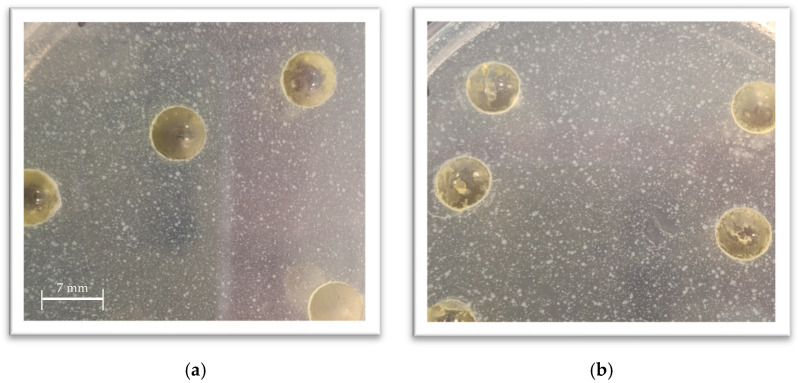
Examples of inhibition zones for microorganisms treated with plant extracts in BSFL fats. Any visible zone has formed. The figure shows *E. coli* treated with cinnamon bark extract (**a**) and rosemary herb extract (**b**).

Appendix A Table A2 provides the exact concentrations used in the study. After determining the MIC for each extract, the following samples were prepared with BSFL fats: 4 × MIC, 2 × MIC, MIC, and MIC/2. The goal was to find out if BSFL fats would be a suitable carrier for plant extracts, facilitating their incorporation into food products and observing any possible synergistic, indifferent, or antagonistic interactions. None of the samples formed an inhibition zone during the test. Figure 2 shows sample photos from antimicrobial efficacy studies with mixtures of plant extracts and BSFL fat. The photographs show that no inhibition zones are formed. Although there are larger and smaller colonies visible on the plate, no significant differences in growth were observed between the control samples prepared without any additional substances and those prepared with BSFL fat only.

These findings bring out a hypothesis, that BSFL fats interact with plant extracts and these interactions lead to lowered antimicrobial activity. Fats and other lipids can act as a carrier for essential oils, helping to stabilize volatile compounds and control their release over time. This can potentially prolong their protective effect within a food matrix, which is a key goal in food preservation.

The antimicrobial efficacy of many SC-CO_2_ extracts compounds is contingent upon their direct interaction with the microbial cell membrane. Consequently, their entrapment within lipid structures, for this instance, in BSFL fats, renders them less accessible for targeting and inhibiting pathogens. Most of the key active compounds in SC-CO_2_ extracts, are lipophilic, meaning they are fat-soluble and have a low affinity for water. In the case of significant amount of BSFL fat, these lipophilic compounds prefer to dissolve in the fat phase rather than the aqueous (water-based) phase, which is surrounding media in Petri dish, or when extrapolated to food matrix—basically most of the foods, containing significant amount of water. And because extracts can so easily mix with BSFL fat, while mixture fills the well in Petri dish, none of the active compounds can diffuse to media and for this reason no inhibition zones are formed. This example shows how many challenges lipophilic substance screening can raise, while having so much insufficiently researched potential in prolongation of food shelf life.

### 2.4. Antibacterial Activity Study in Model Food Matrixes

Since interactions lowering extract antimicrobial properties between high amounts of fats and SC-CO_2_ extracts were noted, further study of SC-CO_2_ extract possibility to provide longer shelf life of food matrix was proceeded with low-fat matrixes of plant (white, wheat-flour based bread) and animal (cow’s milk yoghurt) origin. Empirical studies indicate that SC-CO_2_ extracts displaying significant antimicrobial properties in vitro may necessitate substantially higher concentrations to elicit equivalent effects within high-fat food matrices.

#### 2.4.1. Antibacterial Activity in Animal Origin Food Matrix

When incorporated into food products characterized by elevated fat content, the active compounds within SC-CO_2_ extracts exhibit a tendency to migrate from the aqueous phase into the lipid globules. This process of “partitioning” or “sequestration” within the fat diminishes the concentration of active compounds that are available to engage with and disrupt microbial cells, which are predominantly located in the water-based phase of the food matrix. For this reason, cow’s milk yogurt was chosen as the animal origin matrix, which is very stable in itself and whose fat content is extremely well distributed throughout the product and usually does not change much during storage.

During the study, the total number of microorganisms, yeast and mould growth were monitored. The product was poured into sealed plastic containers and stored for 2 months (59 days), checking at regular intervals not only visually, but also monitoring the changes in the number of selected microorganisms. Detailed results of microorganism growth are presented in Table 3.

The control sample spoiled 17 days after production, as did the samples with DHEx, DCFBEx, CFEx, and RHEx. Since yoghurt naturally contains a large number of live bacteria, the count of total microorganisms was monitored to determine whether the additive had any effect on the lactic acid bacteria. The sample was considered spoiled when yeast or mould growth was observed visually or detected during microbiological testing. The samples with CBEx and GREx remained unspoiled for 59 days, i.e., they were suitable for consumption for 42 days longer than the control sample. The extract content in these samples was very low (0.1%), so it did not affect the color. These results show that SC-CO_2_ extracts have great and still untapped potential to extend the shelf life of food products while ensuring safety.

#### 2.4.2. Antibacterial Activity in Plant Origin Food Matrix

For the experiments with a plant-based matrix, the following plant extracts were used: DHEx, DCFBEx, THEx, RHEx, BLEx, MHEx, ChFEx, CBEx, and GREx. All extracts were added at a concentration of 3%. All samples were prepared as three individual products.

On the control samples (without extract), mold colonies were observed within 4.3 (± 1.5) days. After 65 days, the following samples had no visible mold: those with MHEx (1 out of 3 samples), with CBEx (3 out of 3 samples), with DHEx (3 out of 3 samples), with THEx (3 out of 3 samples), with BLEx (2 out of 3 samples), and with DCFBEx (3 out of 3 samples). One of the samples with MHEx developed visible mold after 15 days, and another after 22 days. One of the BLEx containing samples molded after 6 days of production. The samples that had no visible mold after 65 days were sent for yeast and mold count determination tests, and no yeast or mold colonies were detected in any of the samples.

The findings of this study confirm that certain plant extracts, specifically CBEx, DHEx, THEx, and DCFBEx, are highly effective at inhibiting mold growth in a plant-based food matrix. These extracts offer a promising natural alternative to synthetic preservatives, capable of significantly extending product shelf life and enhancing food safety. While further research is needed to identify the exact active compounds and their minimum inhibitory concentrations, the results clearly support the use of these plant extracts without further fractioning or refining.

#### 2.4.3. Colour Changes Measurement in Plant Origin Food Matrix

The colour of the plant origin food matrix was measured using CIELAB system and results are presented in Table 4. RHEx and THEx contributed to significant changes in color brightness. Compared to the control sample, which had a brightness of 69.95, the RHEx and THEx samples darkened to L values of 49.38 and 39.10, respectively. A negative “a” value indicates a more intense green color, with BLEx, MHEx, DHEx, and THEx contributing most to the intensification of the green color. RHEx shifted the color of the sample towards red, with the “a” value becoming positive at 1.84. A higher positive “b” value indicates a more intense yellow color, and GREx, ChFEx, and DHEx contributed most to the yellow color in the samples. One of the extracts had no significant effect on the color of the product—the samples with DCFBEx L had the smallest difference in a and b values from the control.

## 3. Discussion

The results of this study confirm that SC-CO_2_ botanical extracts possess significant antimicrobial properties, establishing them as promising candidates to be used not only in cosmetics, pharmaceuticals or agriculture, but also in food industry. The efficacy of these extracts was validated in both plant-based and animal–origin food matrices, demonstrating their potential for practical application in food systems. Extracts such as cinnamon (CBEx), dashi (DHEx), thyme (THEx), and clove (DCFBEx) were particularly effective, preventing mold growth in the plant-based matrix for 65 days, a stark contrast to the control samples, which spoiled in less than five days. Similarly, low concentrations of CBEx and GREx extended the shelf life of cow’s milk yogurt by 42 days at concentration as low as 0.1%. These findings are consistent with the existing body of literature, which highlights the antimicrobial activity of compounds like thymol, carvacrol, and cinnamaldehyde, as well as various fatty acids present in the extracts.

Our findings confirm that these extracts possess significant antimicrobial efficacy, successfully extending the shelf life of both animal-based (yogurt) and plant-based food matrices. This performance is largely attributed to the extracts’ rich fatty acid profiles, which contribute to their overall antimicrobial activity alongside essential oil components.

The analysis of MIC demonstrated a wide range of potencies across the selected extracts. RHEx proved to be exceptionally effective, with an MIC of only 0.0156% against *S. aureus* and 0.0313% against *L. monocytogenes*. CBEx also showed high potency with an MIC of 0.0313% against both Gram-positive strains. These results are consistent with the well-documented antimicrobial properties of essential oils from these plants, although our data highlights the specific contribution of fatty acids such as caproic acid (212.6 mg/g in rosemary extract) and capric acid (184.1 mg/g in clove extract) to the total inhibitory effect. While comparable studies on essential oils have shown similar efficacy [18,19,20], our work validates that these less-pure SC-CO_2_ extracts, which are produced through a more sustainable process, are a viable alternative. The research confirms their ability to prolong shelf life in complex food matrices, an area where essential oils alone may not perform as effectively due to their volatile nature.

In a real-world application, the extracts performed exceptionally well. At a low concentration of 0.1% in an animal-based matrix, CBEx and GREx extracts extended the shelf life to 59 days, a significant increase of 42 days compared to the control sample that spoiled in 17 days. Similarly, CBEx, DHEx, THEx, and DCFBEx extracts effectively prevented mould growth in a plant-based matrix for up to 65 days. These results strongly support the use of these natural compounds as effective food preservatives without further refining. However, a key practical consideration is the impact on the sensory qualities of the final product. The addition of THEx and RHEx notably darkened the samples, with their brightness (L) values decreasing to 39.10 and 49.38, respectively, from the control’s 69.95, suggesting a potential for consumer rejection at higher concentrations.

A central hypothesis of this study was that black soldier fly larvae (BSFL) fat, due to its high concentration of lauric acid (464.2 mg/g), would synergistically enhance the antimicrobial effect of the plant extracts. However, our in vitro tests revealed a surprising antagonistic effect. When combined, the extracts and BSFL fat formed no inhibition zones, even at concentrations up to 4 times the MIC. This outcome is likely a result of partitioning, where the lipophilic, active compounds within the extracts are sequestered within the fat phase. This entrapment renders them unable to diffuse into the surrounding aqueous medium to interact with and inhibit pathogens. This finding presents a significant challenge for the food industry and underscores the complex interactions that can occur in multi-component food systems. While BSFL fat could serve a valuable role as a delivery system to control the release of volatile flavour compounds, as our initial hypothesis suggested, its use as a carrier for antimicrobial activity requires a different approach.

A central aspect of this research was the investigation into the interaction between the lipophilic SC-CO_2_ extracts and black soldier fly larvae (BSFL) fat as a potential carrier and a dietary fat substitute. While BSFL fat was anticipated to create a synergistic antimicrobial effect due to its high content of medium-chain fatty acids like lauric acid, the experimental results did not support this hypothesis. Instead, the study observed a significant reduction in antimicrobial efficacy when the extracts were mixed with BSFL fat, as evidenced by the complete absence of inhibition zones in the in vitro tests. This outcome is attributed to the partitioning phenomenon. This phenomena can be quantificated [25] and should be considered in future for further research for industrial applications. The active lipophilic compounds in the extracts, such as terpenes and phenolic compounds, preferentially dissolved and became sequestered within the fat phase rather than migrating to the surrounding aqueous medium to interact with the microorganisms. This phenomenon, which is particularly relevant in high-fat food matrices, demonstrates the critical challenge of ensuring that active compounds are bioavailable at the site of microbial growth.

Future research should focus on mitigating the antagonistic effect observed with BSFL fat. Strategies could include the use of emulsions or other formulation techniques to improve the bioavailability of the extracts’ active compounds within a lipid-rich environment. Further investigation into the precise mechanisms of this partitioning phenomenon could also lead to new methods for designing functional food components that are both sensorily acceptable and technologically effective.

The study of how plant extracts interact with other substances is a significant area of research. While this particular study focused on the interaction of extracts with BSFL fat, other research has explored similar combinations. For instance, a separate study [26] compared the antibacterial effects of zinc oxide (ZnO) nanoparticles combined with thyme and cinnamon essential oils against the pure essential oils alone. The results showed that the mixtures had smaller zones of inhibition (2–43 mm smaller) against bacteria like *L. monocytogenes*, *S. aureus*, and *E. coli*. Conversely, a mixture containing rosemary extract and nanoparticles showed a different result, with inhibition zones that were 1–4 mm larger against the same microorganisms.

Another study [27] explored a different application for similar extracts: creating active packaging by impregnating paper with essential oils. The research focused on minced beef, demonstrating that packaging with oregano essential oil-coated paper significantly reduced microbial growth over a 12-day cold storage period compared to non-coated paper. This packaging also maintained the meat’s colour and sensory qualities at an acceptable level for consumers.

The final assessment, which confirmed the total absence of yeast and mould colonies in the samples that stayed mould-free after 65 days, offers strong proof of the extracts’ effectiveness. This indicates that the extracts not only postponed spoilage but actively suppressed microbial growth to below detectable levels, validating their potential as effective natural preservatives.

## 4. Materials and Methods

SC-CO_2_ (Supercritical Carbon Dioxide) extraction is a widely used method for extracting essential oils, flavourings, and biologically active compounds from plant material. This method exploits the unique properties of CO_2_ when it is in a supercritical state, which occurs above its critical temperature (31.1 °C) and critical pressure (73.8 bar). In this state, CO_2_ exhibits properties of both a gas and a liquid. Before extraction, the plant material is specifically prepared to ensure a better yield and a more effective process. The plant material is dried and ground to increase its surface area, which enhances extraction efficiency. The particle size is optimized to balance maximum surface exposure with minimum flow resistance in the extraction chamber.

The prepared plant material is placed into an extraction vessel, which is typically a stainless-steel chamber capable of withstanding high pressure. CO_2_ is pumped into the extraction vessel, where its pressure is increased to the desired level (in this study, from 300 to 400 bar, depending on the raw material). This pressure allows the CO_2_ to reach its supercritical state. The temperature of the extraction system is maintained between 40 and 60 °C (in this study), depending on the target compounds. A higher temperature can increase solubility but may also degrade sensitive compounds. The supercritical CO_2_ flows through the plant material, dissolving the target compounds. The extraction process can vary in duration, typically lasting from several minutes to several hours, depending on the material and the desired yield.

The pressure of the CO_2_, now containing the dissolved compounds, is then reduced via a separator. As the pressure decreases, the CO_2_ returns to a gaseous state, allowing the soluble substances to precipitate. This process can be precisely tuned to selectively separate different compounds by adjusting the pressure and temperature. The isolated compounds are collected from the separator. The remaining CO_2_ can be captured and recycled back into the extraction system, making the process more sustainable. The products obtained during this extraction include: Essential oils (highly concentrated aromatic compounds), lipophilic compounds (such as fatty acids, sterols, and terpenes), and phytochemicals (including flavonoids, alkaloids, and other biologically active compounds).

The main variables are pressure and temperature. The solubility of compounds in SC-CO_2_ is highly dependent on these parameters. Higher pressure typically increases solubility, while temperature affects the viscosity and density of the CO_2_. The rate at which the CO_2_ is pumped through the extraction vessel can influence extraction efficiency and time, depending on the raw material. A longer extraction time can lead to a higher yield, but it may also extract undesirable compounds. In some cases, small amounts of co-solvents (e.g., ethanol or water) can be added to improve the extraction of specific polar compounds but in this particular study any additional solvents were not used.

All measurements were performed in a manner of at least 3 replicates.

### 4.1. Plant Extract Preparation via Supercritical Carbon Dioxide

Extractions were performed at ŽŪK “Panevėžio Aruodas” (Panevėžys, Lithuania) and delivered for testing immediately after production. Dried herbs were used for extraction and were additionally crushed before extraction. The particle size was optimized to balance maximum surface exposure and minimum flow resistance in the extraction chamber. Extraction was performed using supercritical carbon dioxide, selecting a pressure of 300 to 400 bar and a temperature of 40 to 60 °C in order to achieve the highest possible extract yield and increase commercial potential. All samples of plant extracts obtained were produced by the local company and delivered for research in 10 days after extraction. The following plant extracts were used for the research: caraway seeds, cinnamon bark, rosemary leaves, bay leaves, marjoram herb, thyme herb, ginger root, dried clover flower buds, dashi herb and chamomile flowers. The waxes were removed from extracts by additional winterization.

### 4.2. Lipid Extraction from BSFL

The fat was separated from the total BSF larval biomass by cold pressing as given in detail in our previous work [10]. For this research only pure fats after the pressing and sedimentation process were used. The BSF larvae were grown and their fat was produced by UAB “INSECTUM” (Vilnius, Lithuania) and delivered for testing immediately after production.

### 4.3. Methods of the Microbiological Analysis

#### 4.3.1. Total Number of Microorganisms

The method is based on the ability of mesophilic anaerobic and facultative anaerobic microorganisms (bacteria, yeasts, and moulds) to form colonies in a solid medium at a temperature of 30 ± 1 °C within 72 h. The dilutions of the product to be sown are selected according to the most likely microbial contamination, which in the case of plant extracts and BSFL fats was <10 colony-forming units. For each dilution, take 3 Petri dishes and pour 1 mL of the dilution and 10 mL of medium at a temperature of 45 °C into each dish. Sow three decimals of the dilutions in this way. The amount of product sown is carefully mixed with the medium. For control purposes, plates with medium without the test material are prepared to check sterility. The control and seed plates are inverted and kept for 72 h ± 3 h in a thermostat at a temperature of 30 °C. The number of microorganisms *N* in the test sample is determined by calculating the average of two consecutive dilutions according to the following equation:

This is example 1 of an Equation (1):

(1) $$N=\frac{\sum C}{V\;\times \left[n_{1}+\left(0.1\;\times n_{2}\right)\right]\times d}$$

where

*∑C*—the sum of colonies counted on all non-rejected plates from two consecutive dilutions, when at least one plate contains at least 15 colonies;
*V*—the volume of inoculum on the plate, in milliliters;
*n*_1_—the number of plates evaluated for the first dilution;
*n*_2_—the number of plates evaluated for the second dilution;
*d*—the dilution factor for the first dilution evaluated.

#### 4.3.2. Coliform Count Determination

The determination of individual bacteria was performed as a coliform count at 37 °C, CFU/g, detection of *Salmonella* at 25 g, total number of mesophilic lactic acid bacteria, CFU/g, number of presumptive bifidobacteria, CFU/g, total number of sulphite-reducing bacteria (clostridia), CFU/g, number of yeasts, CFU/g, number of moulds, CFU/g, and detection of monocytogenic listeria (*L. monocytogenes*) at 37 °C at 25 g, using the following methods of analysis: ISO 11133:2014 [28] “Microbiology of food, feed and water”.

#### 4.3.3. *Escherichia coli* Determination

ISO 16649–2:2002 [29] “Microbiology of food and feed—Colony counting in food and feed General method for the enumeration of β–glucuronidase–producing enteric rods (*E. coli*)—Methods and methods Part 2: Methods for the determination of *E. coli* Method for counting colonies at 44 °C using 5–bromo–4–chloro–3–indolyl β–D–glucuronide”.

#### 4.3.4. *Salmonella typhimirium* Determination

Was performed according to ISO 6579-1:2017 [30] “Microbiology of the food chain—Horizontal method for the detection, enumeration and serotyping of *Salmonella* Part 1: Detection of *Salmonella* spp.”.

#### 4.3.5. Enterobacteriaceae Determination

Was performed according to ISO 21528-2:2017 [31] “Microbiology of the food chain—Horizontal method for the detection and enumeration of *Enterobacteriaceae*. Part 2: Colony-count technique”.

#### 4.3.6. *Bacillus cereus* Determination

Was performed according to ISO 7932:2005 [32] “Microbiology of food and animal feeding stuffs—Horizontal method for the enumeration of presumptive *B. cereus*—Colony-count technique at 30 °C”.

#### 4.3.7. *Staphylococcus aureus* Determination

Was performed according to ISO 6888-1:2021 [33], ISO 6888-1:2021/A1:2023.

#### 4.3.8. Yeast and Mould Determination

Was performed according to ISO 21527-1:2008 [34] “Microbiology of food and animal feeding stuffs—Horizontal method for the enumeration of yeasts and moulds. Part 1 and 2: Colony count technique in products with water activity greater than 0.95”.

#### 4.3.9. Antibacterical Activity Determination by Disc Diffusion Method

The agarised media were inoculated with different types of microorganisms (*Bacillus subtilis* ATCC 11778, *S. aureus* ATCC 25923, *E. coli* ATCC 8739, *Salmonella typhimurium* ATCC 14028, *L. monocytogenes* ATCC 13932. On the hardened medium, wells (7 mm in diameter) were cut out and filled with the testing samples of the fat and protein solution (50 µL each well, 6 wells per plate). The plates were incubated with the thermostat at 30 ± 2 °C for *B. cereus* (24 h) and 37 ± 2 °C for *S. aureus*, *E. coli*, *S. typhimurium* and *L. monocytogenes* (24 h). The growth of the isolate was measured with a ruler. The inhibition results are presented as the inhibition coefficient (hereinafter referred to as IC) calculated according to the formula:

IC (%) = *T* × 100*C* (2)

where

C—the size of the control colony, mm.
T—the size of the colony, affected by testing material, mm.

Diameter of measured zones in calculations are being used after extraction of 7 mm, which represents the diameter of well with the tested substance inside.

Bacterial cultures for the testing were grown in 5 mL of brain and heart extract broth at 18 ± 1 °C for 30 h. Bacterial cultures were grown on slanted Tryptone Soy Agar (TSA) for 18–24 h. After incubation, to obtain a bacterial suspension, the washed bacterial suspension was diluted according to McFarlane standard No. 0.5 (1.5 × 10^8^) and mixed thoroughly with a shaker. A 10^−3^ (1.5 × 10^5^) dilution was used for the test. 1 mL of the 10^−3^ dilution was added to 100 mL of TSA medium for bacterial cultures. 10 mL of medium was poured into each plate.

Antibacterial activity is presented as an Inhibition Coefficient (IC). The higher the IC, the greater the extract’s activity. An IC of 0% means that the concentration had no activity, and the microorganism grew the same as in the control sample. An IC of 100% means that at this concentration, not a single culture colony grew during the incubation period. Analyzing the results, it can be observed that there is no linear relationship between the extract concentration and the IC (in not all cases does inhibition strengthen as concentration increases, and vice versa). Such variations are related to the substance’s polarity, volatility, solubility in the chosen solvent, and its ability to diffuse into the medium.

### 4.4. Methods of the Chemical Analysis

#### 4.4.1. Fat Content Determination

Total fat content, % saturated fatty acids, % monounsaturated fatty acids, % polyunsaturated fatty acids, % trans fatty acids, % (LST ISO 1443:197 [35]; LST EN ISO 12966-1:2014 [36]; LST EN ISO 12966-2:2017 [37]). Fatty acid composition, % of total fatty acids: (LST EN ISO 12966-1:2014 LST EN ISO 12966-2:2017).

#### 4.4.2. pH Determination

A 5 g of sample is taken and crushed and added to 25 mL of distilled water, and let it stand for 10 min. After 10 min, the pH value was measured with a calibrated pH meter.

### 4.5. Antimicrobial Activity Testing

#### Agar Diffusion Method

The agar diffusion method is widely used to determine the antibacterial activity of various substances. This method is relatively simple and is based on the diffusion of antimicrobial compounds through a solid agar medium into which bacterial cultures are inoculated. The agar medium was prepared according to the manufacturer’s instructions and sterilized. The inoculum of the selected concentration of bacterial culture was introduced into the sterile agar medium at a temperature not exceeding 30 °C. Using a sterile instrument, wells with a diameter of 7 millimetres were made in the medium (the medium inside the wells was removed). The wells are filled with 50 µL of the prepared test solution (the composition of the solutions is specified in Section 4.7) and, without covering the plate, incubated for 24 h at 30 ± 2 °C for *B. cereus* and 37 ± 2 °C for *S. aureus*, *E. coli*, *S. typhimurium* and *L. monocytogenes*. The inhibition zones are measured in millimetres, according to one selected axis of symmetry. Control samples were also performed for each series of samples by filling the wells with 50 µL of pure (96%) ethyl alcohol or distilled water. Due to the small amount, rapid evaporation and insufficient exposure time in the control samples, ethyl alcohol had no effect on the growth of the culture, therefore any inhibition zones in the extract mixtures with alcohol are considered to be the effect of the plant extract.

### 4.6. Preparation of the Model Food Matrix

#### 4.6.1. Preparation of the Animal Origin Model Food Matrix

Yogurt made from UHT cow’s milk was chosen as the model animal–origin matrix. The control sample was produced from milk and 0.005% commercial yogurt starter culture “DANISCO YO-MIX 883 LYO 50DCU” (“Danisco”, Copenhagen, Denmark), containing *Streptococcus thermophilus* and *Lactobacillus delbueckii* subsp. *bulgaricus*, and carriers—sucrose and maltodextrin. Matrix samples with added plant extract were produced from milk, 0.005% starter culture, and 0.1% extract. The milk is heated to 40 °C and the starter culture and extract are added. The mixture is kept in a 40 °C climate-controlled cabinet until it reaches a pH of 4.6. After the preparation samples were held in separate containers with lids, each weighting 110 g at 4 °C temperature and for each testing separate container was used, to prevent samples from external contamination during storage.

#### 4.6.2. Preparation of the Plant Origin Model Food Matrix

White bread was chosen as the plant model matrix. The control sample was prepared from 60% wheat flour (ash content 0.51–0.63%, gluten content 28–30%) and 40% distilled water. The test samples were prepared from 60% wheat flour, 37% distilled water and 3% extract. The samples were baked at 180 °C for 60 min. Each sample weighed 250 g (±3%) and each combination had 3 separate samples on one run. To preserve moisture, the samples were wrapped in cling film and stored in a place protected from direct sunlight at a temperature of +26.4 (±1.49) °C during the storage period.

### 4.7. Preparation of Testing Substance Mixtures with Ethanol and BSFL Fat

To ensure uniform sample distribution during the determination of antibacterial activity, the extracts were diluted with ethyl alcohol (96%) to 75, 50, 25, 10, 5, 1, 0.5, 0.25, 0.13, 0.063, 0.031, 0.016, 0.0078% of the extract, respectively. A control sample was also performed for each sample series by filling the well with 50 μL of pure (96%) ethyl alcohol. Due to the small amount, rapid evaporation and insufficient exposure time in the control samples, ethyl alcohol had no effect on the growth of the culture, therefore any inhibition zones in the extract mixtures with alcohol are considered to be the effect of the plant extract. The lowest concentration, that showed measurable inhibition to test bacteria growth was considered to be minimal inhibitory concentration (MIC).

### 4.8. Color Characteristic Measurements with CIELAB

The CromaMeter CR-400 (Konica Minolta, Japan) device and the CIELAB system were used to determine the color characteristics, evaluating various parameters (L*—lightness (white); a*—red or −a*—green; b*—yellow or −b*—blue). The colorfulness of the samples was determined by measuring the color characteristics with a Konica Minolta CR–400 colorimeter. The parameters measured were L*, a*, b* (lightness, redness, and yellowness coordinates according to the CIELAB scale, respectively). A standard light source C, whose radiation is close to average daylight, was used for the measurements. The measurements are performed through glass. The measurements are performed in a dark glass, isolated from light distribution, wrapped in a paper cover and placed in a dark container. At least 5 measurements were performed.

### 4.9. Statistical Analysis

GraphPad Prism (ver. 8.0.1) was used for the statistical data processing. A one-way ANOVA test was performed to indicate significant differences where needed.

## 5. Conclusions

This study comprehensively evaluated the potential of SC-CO_2_ plant extracts as natural antimicrobial agents, focusing on their application in various food matrices. The findings conclusively demonstrate that these extracts are a viable and sustainable alternative to conventional preservatives, despite some unique challenges. Our research confirmed that SC-CO_2_ extracts are rich in fatty acids with known antimicrobial properties. For instance, RHEx exhibited exceptional potency, with a MIC of just 0.0156% against *S. aureus* and 0.0313% against *L. monocytogenes*. Its efficacy against *E. coli* was also strong, with an MIC of 1%. Similarly, CBEx showed an MIC of 0.0313% against both *S. aureus* and *L. monocytogenes.*

The antimicrobial activity was directly linked to the fatty acid composition of the extracts. The CBEx contained a high concentration of linoleic acid at 408.8 mg/g, while the DHEx was rich in linolenic acid at 384.7 mg/g. Extracts with significant amounts of capric acid, such as THEx (41.3 mg/g) and DCFBEx (184.1 mg/g), also proved highly effective.

The extracts’ effectiveness was tested in both animal-based and plant-based food matrices. In an animal-based food matrix, CBEx and GREx extracts at a low concentration of 0.1% extended the product’s shelf life by over 42 days, remaining unspoiled for 59 days compared to the control that spoiled in 17 days. In the plant-based matrix, several extracts—specifically CBEx, DHEx, THEx, and DCFBEx—prevented visible mold growth for up to 65 days.

A key consideration, however, was the impact on the final product’s sensory qualities. While highly effective, some extracts caused significant color changes. For example, THEx and RHEx extracts substantially darkened the samples, with their brightness (L) values dropping to 39.10 and 49.38, respectively, compared to the control’s 69.95.

A significant challenge identified was the antagonistic interaction between the plant extracts and BSFL fat, a promising alternative food source. Contrary to the initial hypothesis of synergy, combining the extracts with BSFL fat eliminated all antimicrobial efficacy, with no inhibition zones formed even at concentrations 4 times the MIC. This is attributed to the lipophilic active compounds of the extracts becoming sequestered within the fat, preventing their interaction with microorganisms in the aqueous phase. Despite this challenge, the research’s most significant contribution is the novel exploration of using BSFL fat as a potential delivery system. By using these SC-CO_2_ extracts, which are produced through a more sustainable process, the study provides a foundation for developing technologically advanced and highly effective natural food preservation solutions.

In summary, while SC-CO_2_ extracts are powerful antimicrobial substances, their full potential can only be unlocked by overcoming the bioavailability challenges posed by high-fat environments. Future research should focus on developing innovative delivery systems, such as nano-emulsions or encapsulation techniques, to ensure the active compounds can bypass the lipid phase and maintain their effectiveness in a wide range of food products.

## Figures and Tables

**Table 1:** Fatty acid composition of plant extracts given in mg/g of the extract.

Fatty Acid Name	C:D	Marjoram Herb Extract	Bay Leaf Extract	Dashi Herb Extract	Cinnamon Bark Extract	Rosemary Herb Extract	Thyme Herb Extract	Caraway Fruit Extract	Chamomile Flower Extract	Ginger Root Extract	Dried Clove Flower Bud Extract
Extract Name Abbreviation		MHEx	BLEx	DHEx	CBEx	RHEx	THEx	CFEx	ChFEx	GREx	DCFBEx
Caproic acid	C 6:0	5.2	0.0	6.1	1.1	212.6	25.2	0.0	0.0	1.6	0.0
Caprylic acid	C 8:0	1.6	0.0	0.0	1.4	0.0	0.0	0.0	0.0	0.0	1.6
Capric acid	C 10:0	2.8	0.0	0.1	0.5	21.0	41.3	0.1	24.7	0.0	184.1
Undecylic acid	C 11:0	59.3	15.9	0.0	0.0	0.0	6.2	0.0	0.0	10.5	0.0
Lauric acid	C 12:0	0.0	29.6	0.0	0.0	19.8	0.0	0.0	0.0	0.0	0.0
Myristic acid	C 14:0	0.0	2.1	0.0	0.2	0.0	0.0	0.0	0.0	0.0	119.6
Myristoleic acid	C 14:1	0.0	0.0	0.0	0.2	15.2	0.0	0.0	0.0	0.0	0.0
Pentadecanoic acid	C 15:0	0.0	0.0	0.0	0.0	0.0	0.0	0.0	6.0	0.0	0.0
Palmitic acid	C 16:0	19.3	70.2	27.5	7.1	74.6	42.7	15.3	39.0	57.2	23.4
Palmitoleic acid	C 16:1	0.0	30.4	1.6	3.8	16.7	8.7	0.3	16.7	17.4	29.0
Margaric acid	C 17:0	0.0	15.6	0.2	0.8	0.0	1.3	0.0	45.3	0.0	0.4
Heptadecenoic acid	C 17:1	0.0	0.7	0.0	0.0	1.1	18.5	0.0	0.0	0.0	0.0
Stearic acid	C 18:0	2.3	10.4	9.5	1.2	10.7	13.4	4.3	8.3	13.7	5.5
Elaidic acid	C 18:1 tr	0.0	0.0	2.1	0.0	0.0	0.0	0.0	0.0	0.0	0.0
Oleic acid	C 18:1	19.0	61.8	44.3	6.5	62.9	35.3	215.4	76.0	48.7	20.5
Trans-linoleic acid	C 18:2 tr	0.0	2.7	2.2	0.0	6.8	8.7	0.1	21.2	0.0	0.0
Linoleic acid	C 18:2	24.6	89.4	175.7	408.8	39.4	57.2	119.5	68.4	56.3	20.4
Arachidic acid	C 20:0	1.0	48.7	0.0	1.1	43.0	2.9	0.1	1.5	24.9	1.4
Gamma-Linolenic acid	C 18:3g	4.2	39.6	1.5	3.6	3.7	27.3	0.0	264.5	7.0	0.0
Gadoleic acid	C 20:1	0.0	0.0	0.0	0.0	0.0	0.0	0.3	0.0	0.0	0.0
Alpha-Linolenic acid	C 18:3a	54.2	170.5	384.7	7.2	25.1	95.9	0.8	21.0	30.0	4.4
Heneicosanoic acid	C 21:0	9.7	12.1	10.7	0.4	19.1	0.0	0.9	9.9	5.3	3.7
Eicosadienoic acid	C 20:2	0.0	0.0	0.0	5.1	0.0	0.0	0.0	0.1	0.0	0.0
Behenic acid	C 22:0	2.0	8.8	0.0	0.0	0.0	6.7	0.0	3.6	3.3	0.0
Dihomo-gamma-linolenic acid	C 20:3w6	0.0	0.0	0.0	0.0	0.0	7.0	0.0	2.7	0.0	0.0
Eicosatrienoic acid	C 20:3w3	7.0	14.9	0.5	1.3	40.2	12.0	0.8	0.0	0.0	4.8
Arachidonic acid	C 20:4	0.0	1.5	0.0	0.0	1.3	0.0	0.0	0.0	0.0	0.0
Tricosanoic acid	C 23:0	0.0	0.0	0.0	4.6	0.0	0.0	0.0	0.0	0.0	21.1
Docosadienoic acid	C 22:2	0.0	0.0	0.0	0.0	1.6	0.0	0.0	1.3	0.0	0.0
Lignoceric acid	C 24:0	7.5	30.1	0.1	0.0	2.5	9.7	0.0	0.0	3.8	0.0
Eicosapentaenoic acid (EPA)	C 20:5	4.3	8.9	0.0	0.0	2.7	10.9	0.0	0.0	0.0	0.0
Nervonic acid	C 24:1	0.0	0.0	0.0	0.0	0.0	2.6	0.0	0.0	0.0	0.0

**Table 2:** Minimal inhibition concentrations based on inhibition coefficient of ethanolic solutions of plant extracts.

Used Extract	MIC, %		
	ATCC 8739 *E. coli*	ATCC 25923 *S. aureus*	ATCC 13932 *L. monocytogenes*
THEx	0.125	0.0625	0.0625
ChFEx	>75	0.125	0.500
BLEx	50	0.500	0.125
DHEx	5	0.0625	0.125
RHEx	1	0.0156	0.0313
CBEx	0.500	0.0313	0.0313
MHEx	5	0.25	0.0625
DCFBEx	0.25	0.25	0.125
GREx	>75	0.0625	1
CFEx	>75	1	1

**Table 3:** Microorganism growth during storage of animal origin food matrix.

Days After Production	3	10	17	24	31	45	59
Control							
Total microorganism count, log CFU/g	8.48	4.53	8.43	7.99	SP	SP	SP
Total mould count, log CFU/g	0.00	0.00	4.93	5.38	SP	SP	SP
Total yeast count, log CFU/g	0.00	3.11	2.08	3.30	SP	SP	SP
Dashi herb extract							
Total microorganism count, CFU/g	8.45	5.08	8.41	8.41	SP	SP	SP
Total mould count, CFU/g	0.00	0.00	5.56	6.28	SP	SP	SP
Total yeast count, CFU/g	0.00	4.54	0.00	0.00	SP	SP	SP
Dried clove flower bud extract							
Total microorganism count, CFU/g	8.48	4.04	8.43	9.23	SP	SP	SP
Total mould count, CFU/g	0.00	0.00	3.04	0.00	SP	SP	SP
Total yeast count, CFU/g	0.00	3.15	0.00	0.00	SP	SP	SP
Caraway fruit extract							
Total microorganism count, CFU/g	8.38	6.57	7.43	8.34	SP	SP	SP
Total mould count, CFU/g	0.00	0.00	7.04	6.65	SP	SP	SP
Total yeast count, CFU/g	0.00	6.08	0.00	0.00	SP	SP	SP
Rosemary herb extract							
Total microorganism count, CFU/g	8.23	5.43	8.23	7.75	SP	SP	SP
Total mould count, CFU/g	0.00	0.00	5.81	5.87	SP	SP	SP
Total yeast count, CFU/g	0.78	4.43	0.00	0.00	SP	SP	SP
Marjoram herb extract							
Total microorganism count, CFU/g	8.45	4.11	8.41	7.94	8.52	SP	SP
Total mould count, CFU/g	0.00	0.00	0.00	4.58	0.00	SP	SP
Total yeast count, CFU/g	0.00	2.15	0.00	0.00	5.62	SP	SP
Chamomile flower extract							
Total microorganism count, CFU/g	8.48	6.52	8.46	8.51	8.18	4.11	SP
Total mould count, CFU/g	0.00	0.00	0.00	0.00	0.00	0.00	SP
Total yeast count, CFU/g	0.00	0.00	0.00	0.00	0.00	0.00	SP
Cinnamon bark extract							
Total microorganism count, CFU/g	6.73	2.08	5.36	5.54	5.57	5.83	6.23
Total mould count, CFU/g	0.00	0.00	0.00	0.00	0.00	0.00	0.00
Total yeast count, CFU/g	0.00	0.00	0.00	0.00	0.00	0.00	0.00
Ginger root extract							
Total microorganism count, CFU/g	8.48	3.68	8.48	8.48	8.18	3.26	6.11
Total mould count, CFU/g	0.00	0.00	0.00	0.00	0.00	0.00	0.00
Total yeast count, CFU/g	0.00	0.00	0.00	0.00	0.00	0.00	0.00

SP—spoiled.

**Table 4:** Results of color measurement using CIELAB system.

Colour Characteristic	Control	MHEx	CBEx	RHEx	DHEx
L*	69.95 ± 0.58	52.32 ± 0.09	60.52 ± 1.17	49.38 ± 1.36	50.46 ± 0.85
a*	−1.45 ± 0.07	−4.73 ± 0.14	−2.28 ± 0.10	1.84 ± 0.38	−4.67 ± 0.16
b*	19.10 ± 0.38	34.34 ± 0.40	33.58 ± 0.33	28.87 ± 0.18	43.58 ± 0.57
h, degree	−1.50 ± 0.00	−1.43 ± 0.01	−1.50 ± 0.00	1.51 ± 0.01	−1.46 ± 0.00
C	19.16 ± 0.38	34.67 ± 0.38	33.65 ± 0.34	28.93 ± 0.18	43.83 ± 0.58
**Colour Characteristic**	**THEx**	**ChFEx**	**BLEx**	**DCFBEx**	**GREx**
L*	39.10 ± 0.24	60.35 ± 0.40	59.45 ± 0.57	69.70 ± 0.52	53.53 ± 1.05
a*	−3.24 ± 0 06	−3.25 ± 0.12	−5.76 ± 0.10	−2.16 ± 0.07	−4.68 ± 0.11
b*	28.77 ± 0.44	42.60 ± 1.20	38.31 ± 0.26	30.97 ± 0.57	42.35 ± 0.50
h, degree	−1.46 ± 0.00	−1.49 ± 0.00	−1.42 ± 0.00	−1.50 ± 0.00	−1.46 ± 0.00
C	28.95 ± 0.44	42.72 ± 1.19	38.74 ± 0.26	31.04 ± 0.57	42.61 ± 0.51

## Data Availability

The original contributions presented in this study are included in the article. Further inquiries can be directed to the corresponding author.

## Abbreviations

The following abbreviations are used in this manuscript:

BSF	Black soldier fly
BSFL	Black soldier fly larvae
IC	Inhibition coefficient
TSA	Tryptone Soy Agar
MIC	Minimal inhibitory concentration
ND	Not detected
SP	Spoiled
SC-CO_2_	Super critical carbon dioxide
MHEx	Marjoram herb extract
BLEx	Bay leaf extract
DHEx	Dashi herb extract
CBEx	Cinnamon bark extract
RHEx	Rosemary herb extract
THEx	Thyme herb extract
CFEx	Caraway fruit extract
ChFEx	Chamomile flower extract
GREx	Ginger root extract
DCFBEx	Dried clove flower bud extract

## Appendix A

Additional data and a complete summary of the conducted studies are presented here, supplementing the most significant results provided in the manuscript text. These tables contain some repetitive information, but they are presented in this format to facilitate data review for the reader.

**Table A1:** Full data on minimal inhibitory concentration testing.

Used Extract	Concentration, %	Inhibition Coefficient, %		
		ATCC 8739 *E. coli*	ATCC 25923 *S. aureus*	ATCC 13932 *L. monocytogenes*
Thyme herb extract	75	78.4	100.0	100.0
	50	93.2	100.0	100.0
	25	78.4	100.0	100.0
	10	70.3	100.0	100.0
	5	75.7	100.0	100.0
	1	58.1	100.0	100.0
	0.5	12.2	34.4	21.1
	0.25	10.0	22.2	14.4
	0.125	6.7	14.4	10.0
	0.0625	0.0	14.4	6.7
	0.03125	0.0	0.0	0.0
Chamomile flower extract	75	Sample was too thick to dose properly		
	50	0.0	70.3	47.3
	25	0.0	67.6	36.5
	10	0.0	50.0	37.8
	5	0.0	73.0	43.2
	1	0.0	54.1	55.4
	0.5	0.0	14.4	3.3
	0.25	0.0	13.3	0.0
	0.125	0.0	10.0	0.0
	0.0625	0.0	0.0	0.0
	0.03125	0.0	0.0	0.0
Bay leaf extract	75	0.0	68.9	45.9
	50	31.1	81.1	58.1
	25	0.0	73.0	55.4
	10	0.0	62.2	39.2
	5	0.0	56.8	47.3
	1	0.0	31.1	21.6
	0.5	0.0	14.4	6.7
	0.25	0.0	0.0	2.2
	0.125	0.0	0.0	2.2
	0.0625	0.0	0.0	0.0
	0.03125	0.0	0.0	0.0
Dashi herb extract	75	37.8	100.0	100.0
	50	71.6	100.0	100.0
	25	51.4	100.0	100.0
	10	45.9	100.0	100.0
	5	74.3	100.0	100.0
	1	0.0	100.0	100.0
	0.5	0.0	26.7	45.6
	0.25	0.0	17.8	42.2
	0.125	0.0	12.2	10.0
	0.0625	0.0	6.7	0.0
	0.03125	0.0	0.0	0.0
Rosemary herb extract	75	0.0	100.0	62.2
	50	36.5	100.0	77.0
	25	48.6	100.0	70.3
	10	40.5	100.0	51.4
	5	43.2	100.0	64.9
	1	28.4	100.0	54.1
	0.5	0.0	56.7	46.7
	0.25	0.0	56.7	36.7
	0.125	0.0	38.9	28.9
	0.0625	0.0	26.7	21.1
	0.03125	0.0	25.0	14.4
	0.0156	0.0	13.3	0.0
	0.0078	0.0	0.0	0.0
Cinnamon bark extract	75	100.0	100.0	100.0
	50	100.0	100.0	100.0
	25	100.0	100.0	100.0
	10	100.0	100.0	100.0
	5	100.0	100.0	100.0
	1	100.0	100.0	100.0
	0.5	22.2	46.7	24.4
	0.25	0.0	24.4	14.4
	0.125	0.0	14.4	11.1
	0.0625	0.0	10.0	10.0
	0.03125	0.0	7.8	6.7
	0.0156	0.0	0.0	0.0
	0.0078	0.0	0.0	0.0
Marjoram herb extract	75	50.0	100.0	63.5
	50	59.5	100.0	86.5
	25	54.1	100.0	66.2
	10	41.9	100.0	56.8
	5	41.9	100.0	73.0
	1	0.0	100.0	43.2
	0.5	0.0	27.8	15.6
	0.25	0.0	15.6	14.4
	0.125	0.0	0.0	6.7
	0.0625	0.0	0.0	4.4
	0.03125	0.0	0.0	0.0
Dried clove flower bud extract	75	59.5	67.6	56.8
	50	64.9	73.0	62.2
	25	51.4	64.9	51.4
	10	27.0	64.9	24.3
	5	62.2	62.2	54.1
	1	16.2	33.8	37.8
	0.5	13.3	22.2	12.2
	0.25	6.7	8.9	6.7
	0.125	0.0	0.0	3.3
	0.0625	0.0	0.0	0.0
	0.03125	0.0	0.0	0.0
Ginger root extract	75	0.0	37.8	24.3
	50	0.0	54.1	33.8
	25	0.0	45.9	32.4
	10	0.0	44.6	35.1
	5	0.0	64.9	43.2
	1	0.0	35.1	37.8
	0.5	0.0	36.7	0.0
	0.25	0.0	27.8	0.0
	0.125	0.0	17.8	0.0
	0.0625	0.0	11.1	0.0
	0.03125	0.0	0.0	0.0
Caraway fruit extract	75	0.0	100.0	40.5
	50	0.0	100.0	48.6
	25	0.0	100.0	43.2
	10	0.0	100.0	31.1
	5	0.0	100.0	37.8
	1	0.0	100.0	24.3
	0.5	0.0	0.0	0.0
	0.25	0.0	0.0	0.0
	0.125	0.0	0.0	0.0
	0.0625	0.0	0.0	0.0
	0.03125	0.0	0.0	0.0

**Table A2:** Full data on synergistic activity with BSFL fat testing.

	ATCC 25923 *S. aureus*				ATCC 13932 *L. monocytogenes*				ATCC 8739 *E. coli*			
	×4	×2	MIC	/2	×4	×2	MIC	/2	×4	×2	MIC	/2
Thyme herb extract	0.250	0.125	0.063	0.031	0.250	0.125	0.063	0.031	0.500	0.250	0.125	0.063
Chamomile flower extract	0.500	0.250	0.125	0.063	2.000	1.000	0.500	0.250	MIC > 1%			
Bay leaf extract	2.000	1.000	0.500	0.250	0.500	0.250	0.125	0.063	MIC > 1%			
Dashi herb extract	0.250	0.125	0.063	0.031	0.500	0.250	0.125	0.063	MIC > 1%			
Rosemary herb extract	0.063	0.031	0.016	0.008	0.125	0.063	0.031	0.016	4.000	2.000	1.000	0.500
Cinnamon bark extract	0.125	0.063	0.031	0.016	0.125	0.063	0.031	0.016	2.000	1.000	0.500	0.250
Marjoram herb extract	1.000	0.500	0.250	0.125	0.250	0.125	0.063	0.031	MIC > 1%			
Dried clove flower bud extract	1.000	0.500	0.250	0.125	0.500	0.250	0.125	0.063	1.000	0.500	0.250	0.125
Ginger root extract	0.250	0.125	0.063	0.031	4.000	2.000	1.000	0.500	0.250	0.125	0.063	0.031
Caraway fruit extract	MIC > 1%				4.000	2.000	1.000	0.500	MIC > 1%			

**Table A3:** Full data on extract sterility testing.

Title 1	DHEx	DCFBEx	CFEx	RHEx	RHEx	BLEx	MHEx	ChFEx	CBEx	GREx	Method
Total microorganism count KSV/g	<1.0	<1.0	<1.0	<1.0	<1.0	<1.0	<1.0	<1.0	<1.0	<1.0	4.3.1.
Number of β-glucuronidase-producing *E. coli*, CFU/g	<1.0	<1.0	<1.0	<1.0	<1.0	<1.0	<1.0	<1.0	<1.0	<1.0	4.3.3.
Detection of coliform bacteria at 37 °C, 1 g	ND	ND	ND	ND	ND	ND	ND	ND	ND	ND	4.3.2.
Count of Enterobacteria (*Enterobacteriaceae*) at 37 °C, CFU/g	<1.0	<1.0	<1.0	<1.0	<1.0	<1.0	<1.0	<1.0	<1.0	<1.0	4.3.5.
Count of presumptive *Bacillus cereus* at 30 °C, CFU/g	<1.0	<1.0	<1.0	<1.0	<1.0	<1.0	<1.0	<1.0	<1.0	<1.0	4.3.6.
Count of coagulase-positive staphylococci (*S. aureus* and other species) at 37 °C, CFU/g	<1.0	<1.0	<1.0	<1.0	<1.0	<1.0	<1.0	<1.0	<1.0	<1.0	4.3.7.
Count of *L. monocytogenes* at 37 °C, CFU/g	<1.0	<1.0	<1.0	<1.0	<1.0	<1.0	<1.0	<1.0	<1.0	<1.0	4.3.2.
Detection of Salmonella, 10 g	ND	ND	ND	ND	ND	ND	ND	ND	ND	ND	4.3.4.
Yeast count, CFU/g	<1.0	<1.0	<1.0	<1.0	<1.0	<1.0	<1.0	<1.0	<1.0	<1.0	4.3.8.
Mold count, CFU/g	<1.0	<1.0	<1.0	<1.0	<1.0	<1.0	<1.0	<1.0	<1.0	<1.0	4.3.8.

## References

[1] Hofmeisterová L., Bajer T., Walczak M., Šilha D. (2024). Chemical Composition and Antibacterial Effect of Clove and Thyme Essential Oils on Growth Inhibition and Biofilm Formation of Arcobacter spp. and Other Bacteria. Antibiotics.

[2] Das S., Horváth B., Šafranko S., Jokić S., Széchenyi A., Kőszegi T. (2019). Antimicrobial Activity of Chamomile Essential Oil: Effect of Different Formulations. Molecules.

[3] Siriken B., Yavuz C., Guler A. (2018). Antibacterial Activity of Laurus nobilis: A review of literature. Med. Sci. Discov..

[4] Cagal M.M., Taner G., Kalaycı S., Duman G. (2025). Enhanced antibacterial and genoprotective properties of nanoliposomal *Satureja hortensis* L. essential oil. Drug Chem. Toxicol..

[5] Olivas-Méndez P., Chávez-Martínez A., Santellano-Estrada E., Asorey L.G., Sánchez-Vega R., Rentería-Monterrubio A.L., Chávez-Flores D., Tirado-Gallegos J.M., Méndez-Zamora G. (2022). Antioxidant and Antimicrobial Activity of Rosemary (*Rosmarinus officinalis*) and Garlic (*Allium sativum*) Essential Oils and Chipotle Pepper Oleoresin (*Capsicum annum*) on Beef Hamburgers. Foods.

[6] Shan B., Cai Y.-Z., Brooks J.D., Corke H. (2007). Antibacterial Properties and Major Bioactive Components of Cinnamon Stick (*Cinnamomum burmannii*):  Activity against Foodborne Pathogenic Bacteria. J. Agric. Food Chem..

[7] Walker J.F., Santos P.D.S., Schmidt C.A., Bittencourt T.C.C.D., Guimarães A.G. (2016). Antimicrobial Activity of Marjoram (*Origanum majorana*) Essential Oil Against the Multidrug-Resistant *Salmonella enterica* Serovar Schwarzengrund Inoculated in Vegetables from Organic Farming. J. Food Saf..

[8] Abdullahi A., Khairulmazmi A., Yasmeen S., Ismail I.S., Norhayu A., Sulaiman M.R., Ahmed O.H., Ismail M.R. (2020). Phytochemical profiling and antimicrobial activity of ginger (*Zingiber officinale*) essential oils against important phytopathogens. Arab. J. Chem..

[9] Wanner J., Bail S., Buchbauer G., Schmidt E., Gochev V., Girova T., Atanasova T., Stoyanova A. (2010). Chemical Composition and Antimicrobial Activity of Cumin Oil (*Cuminum cyminum*, Apiaceae). Nat. Prod. Commun..

[10] Zabulionė A., Šalaševičienė A., Makštutienė N., Šarkinas A. (2023). Exploring the Antimicrobial Potential and Stability of Black Soldier Fly (*Hermentia illucens*) Larvae Fat for Enhanced Food Shelf-Life. Gels.

[11] Belguesmia Y., Rabesona H., Mounier J., Pawtowsky A., Le Blay G., Barbier G., Haertlé T., Chobert J.-M. (2014). Characterization of antifungal organic acids produced by *Lactobacillus harbinensis* K.V9.3.1Np immobilized in gellan–xanthan beads during batch fermentation. Food Control.

[12] Antoce O.-A., Antoce V., Pomohaci N., Namolosanu I., Takahashi K. (1998). Inhibitory Effect of Decanoic Acid on Yeast Growth at Various pHs and Ethanol Concentrations. Biocontrol Sci..

[13] Jin X., Zhou J., Richey G., Wang M., Hong S.M.C., Hong S.H. (2021). Undecanoic Acid, Lauric Acid, and N-Tridecanoic Acid Inhibit *Escherichia coli* Persistence and Biofilm Formation. J. Microbiol. Biotechnol..

[14] Ammendola S., Lembo A., Battistoni A., Tagliatesta P., Ghisalberti C., Desideri A. (2009). 10-Undecanhydroxamic acid, a hydroxamate derivative of the undecanoic acid, has strong antimicrobial activity through a mechanism that limits iron availability. FEMS Microbiol. Lett..

[15] Casillas-Vargas G., Ocasio-Malavé C., Medina S., Morales-Guzmán C., Del Valle R.G., Carballeira N.M., Sanabria-Ríos D.J. (2021). Antibacterial fatty acids: An update of possible mechanisms of action and implications in the development of the next-generation of antibacterial agents. Prog. Lipid Res..

[16] Greenway D.L.A., Dyke K.G.H. (1979). Mechanism of the Inhibitory Action of Linoleic Acid on the Growth of *Staphylococcus aureus*. J. Gen. Microbiol..

[17] Park K., Mok J.S., Kwon J.Y., Ryu A.R., Kim S.H., Lee H.J. (2018). Food-borne outbreaks, distributions, virulence, and antibiotic resistance profiles of Vibrio parahaemolyticus in Korea from 2003 to 2016: A review. Fish. Aquat. Sci..

[18] Kabotso D.E.K., Neglo D., Gaba S.E., Danyo E.K., Dayie A.D., Asantewaa A.A., Kotey F.C.N., Dayie N.T.K.D. (2024). In Vitro Evaluation of Rosemary Essential Oil: GC-MS Profiling, Antibacterial Synergy, and Biofilm Inhibition. Pharmaceuticals.

[19] Sethunga M., Ranasinghe M.M.K.D., Ranaweera K.K.D.S., Munaweera I., Gunathilake K.D.P.P. (2023). Synergistic antimicrobial activity of essential oils and oleoresins of cinnamon (*Cinnamomum zeylanicum*), clove bud (*Syzygium aromaticum*) and ginger (*Zingiber officinale*). Biocatal. Agric. Biotechnol..

[20] Mráz P., Kopecký M., Hasoňová L., Hoštičková I., Vaníčková A., Perná K., Žabka M., Hýbl M. (2025). Antibacterial Activity and Chemical Composition of Popular Plant Essential Oils and Their Positive Interactions in Combination. Molecules.

[21] Vassiliou E., Awoleye O., Davis A., Mishra S. (2023). Anti-Inflammatory and Antimicrobial Properties of Thyme Oil and Its Main Constituents. Int. J. Mol. Sci..

[22] Paudel P.N., Satyal P., Satyal R., Setzer W.N., Gyawali R. (2022). Chemical Composition, Enantiomeric Distribution, Antimicrobial and Antioxidant Activities of *Origanum majorana* L. Essential Oil from Nepal. Molecules.

[23] Abu Ghazal T.S., Schelz Z., Vidács L., Szemerédi N., Veres K., Spengler G., Hohmann J. (2022). Antimicrobial, Multidrug Resistance Reversal and Biofilm Formation Inhibitory Effect of *Origanum majorana* Extracts, Essential Oil and Monoterpenes. Plants.

[24] Ivanovic J., Misic D., Ristic M., Pesic O., Zizovic I. (2010). Supercritical CO_2_ extract and essential oil of bay (*Laurus nobilis* L.): Chemical composition and antibacterial activity. J. Serbian Chem. Soc..

[25] Costa M., Losada-Barreiro S., Paiva-Martins F., Bravo-Díaz C. (2021). Polyphenolic Antioxidants in Lipid Emulsions: Partitioning Effects and Interfacial Phenomena. Foods.

[26] Motelica L., Vasile B.-S., Ficai A., Surdu A.-V., Ficai D., Oprea O.-C., Andronescu E., Mustățea G., Ungureanu E.L., Dobre A.A. (2023). Antibacterial Activity of Zinc Oxide Nanoparticles Loaded with Essential Oils. Pharmaceutics.

[27] Yasar S., Nizamlıoğlu N.M., Gücüş M.O., Dal A.E.B., Akgül K. (2022). *Origanum majorana* L. Essential Oil-Coated Paper Acts as an Antimicrobial and Antioxidant Agent against Meat Spoilage. ACS Omega.

[28] (2014). Microbiology of Food, Animal Feed and Water-Preparation, Production, Storage and Performance Testing of Culture Media.

[29] (2002). Microbiology of Food and Animal Feeding Stuffs-Horizontal Method for the Enumeration of Beta-Glucuronidase-Positive *Escherichia coli*-Part 2: Colony-Count Technique at 44 °C Using 5-Bromo-4-Chloro-3-Indolyl Beta-D-Glucuronide.

[30] (2017). Microbiology of the Food Chain-Horizontal Method for the Detection, Enumeration and Serotyping of Salmonella-Part 1: Detection of *Salmonella* spp..

[31] (2017). Microbiology of the Food Chain-Horizontal Method for the Detection and Enumeration of Enterobacteriaceae-Part 2: Colony-Count Technique.

[32] (2005). Microbiology of Food and Animal Feeding Stuffs-Horizontal Method for the Enumeration of Presumptive Bacillus Cereus-Colony-Count Technique at 30 °C.

[33] (2021). Microbiology of the Food Chain-Horizontal Method for the Enumeration of Coagulase-Positive Staphylococci (*Staphylococcus aureus* and Other Species)-Part 1: Detection and Enumeration Using Baird-Parker Agar with Egg Yolk Tellurite Supplement.

[34] (2008). International Organization for Standardization. Microbiology of Food and Animal Feeding Stuffs-Horizontal Method for the Enumeration of Yeasts and Moulds-Part 1: Colony Count Technique in Products with Water Activity Greater than 0.95.

[35] (2000). Meat and Meat Products-Determination of Total Fat Content.

[36] (2014). Animal and Vegetable Fats and Oils-Gas Chromatography of Fatty Acid Methyl Esters-Part 1: Guidelines on Modern Gas Chromatography of Fatty Acid Methyl Esters.

[37] (2017). Animal and Vegetable Fats and Oils-Gas Chromatography of Fatty Acid Methyl Esters-Part 2: Preparation of Methyl Esters of Fatty Acids.

